# Super-*Agrobacterium* ver. 4: Improving the Transformation Frequencies and Genetic Engineering Possibilities for Crop Plants

**DOI:** 10.3389/fpls.2019.01204

**Published:** 2019-10-07

**Authors:** Satoko Nonaka, Tatsuhiko Someya, Yasuhiro Kadota, Kouji Nakamura, Hiroshi Ezura

**Affiliations:** ^1^Tsukuba Plant Innovation Research Center, Gene Research Center, University of Tsukuba, Tsukuba, Japan; ^2^Faculty of Life and Environmental Sciences, University of Tsukuba, Tsukuba, Japan; ^3^RIKEN Center for Sustainable Resource Science, Plant Immunity Group, Yokohama, Japan

**Keywords:** *Agrobacterium tumefaciens*, Super-*Agrobacterium*, genetic engineering, plant transformation, gamma-aminobutyric acid, ethylene, GABA transaminase, ACC deaminase

## Abstract

*Agrobacterium tumefaciens* has been utilized for both transient and stable transformations of plants. These transformation methods have been used in fields such as breeding GM crops, protein production in plant cells, and the functional analysis of genes. However, some plants have significantly lower transient gene transfer and stable transformation rates, creating a technical barrier that needs to be resolved. In this study, Super-*Agrobacterium* was updated to ver. 4 by introducing both the ACC deaminase (*acdS*) and GABA transaminase (*gabT*) genes, whose resultant enzymes degrade ACC, the ethylene precursor, and GABA, respectively. *A. tumefaciens* strain GV2260, which is similar to other major strains (EHA105, GV3101, LBA4404, and MP90), was used in this study. The abilities of the Super-*Agrobacterium* ver. 4 were evaluated in *Erianthus ravennae*, *Solanum lycopersicum* “Micro-Tom,” *Nicotiana benthamiana*, and *S. torvum*. Super-*Agrobacterium* ver. 4 showed the highest T-DNA transfer (transient transformation) frequencies in *E. ravennae* and *S. lycopersicum*, but not in *N. benthamiana* and *S. torvum*. In tomato, Super-*Agrobacterium* ver. 4 increased the stable transformation rate by 3.6-fold compared to the original GV2260 strain. Super-*Agrobacterium* ver. 4 enables reduction of the amount of time and labor required for transformations by approximately 72%, and is therefore a more effective and powerful tool for plant genetic engineering and functional analysis, than the previously developed strains. As our system has a plasmid containing the *acdS* and *gabT* genes, it could be used in combination with other major strains such as EHA105, EHA101, LBA4404, MP90, and AGL1. Super-*Agrobacterium* ver. 4, could thus possibly be a breakthrough application for improving basic plant science research methods.

## Introduction

*Agrobacterium tumefaciens* is an α-proteobacteria that causes crown gall disease in many agriculturally and economically important species, such as those from the families *Rosaceae* (rose, apple, cherry, and pear), *Vitaceae* (grape), and the genus Juglans (walnut) (Kado, 2014). *A. tumefaciens* has the ability to transfer T-DNA from bacterial cells to plant cells (T-DNA transfer). Transferred T-DNA is integrated into the plant genome *via* complicated plant cell systems (Guo et al., 2019), and results in crown gall disease. To utilize this unique ability of *A. tumefaciens* for research purposes, there has been a great deal of effort to remove its oncogenesis characteristics, and to develop a binary vector system (Zambryski et al., 1983; Hoekema et al., 1983; Bevan, 1984; Komari et al., 2006). There has been further effort to increase the T-DNA transfer frequency of *A. tumefaciens*; one effective strategy was to upregulate its *vir* gene expression levels. The application of *vir* gene inducers (Stachel et al., 1985; Stachel et al., 1986; Cangelosi et al., 1990; He et al., 2009; Hu et al., 2013), using ternary system (van der Fits et al., 2000), utilization of Super-binary vectors (Komari, 1990; Hiei et al., 1994; Ishida et al., 1996), and a modification of the Ori of the binary vector (Ye et al., 2011; Vaghchhipawala et al., 2018), have subsequently improved its transformation frequencies.

Another strategy to increase T-DNA transfer frequencies was the removal of the negative factors of the *Agrobacterium*-plant interactions, such as ethylene, the gaseous phytohormone. Applications, such as aminoethoxyvinylglycine (AVG), an ethylene biosynthesis inhibitor, and AgNO_3_ or silver thiosulfate (STS), ethylene perception inhibitors, were effective at improving the T-DNA transfer frequencies in tomato, melon, and bottle gourd (Davis et al., 1992; Ezura et al., 2000; Han et al., 2005; Nonaka and Ezura, 2014). An alternative strategy was the utilization of ACC deaminase (AcdS) activity, which cleaves ACC, the ethylene precursor, to ammonia and α keto-butyrate. This enzyme was found in some plant growth promoting bacteria (PGPBs), such as *Pseudomonas* sp., which were found on the plant surface (Sheehy et al., 1991), and these bacteria utilize ACC as a nitrogen source. However, *A. tumefaciens* C58 strain, which was the original strain for *Agrobacterium*-mediated transformation, does not have the *acdS* gene or its activities (Wood et al., 2001; Nonaka et al., 2008a). Therefore, the utilization AcdS activity seemed to be reasonable. Indeed, *A. tumefaciens* GV2260 that had AcdS activity introduced into it, was efficacious in the suppression of ethylene evolution from plant tissues during co-cultivation and increasing T-DNA transfer [Nonaka et al., 2008a (Super-*Agrobacterium* ver.1); Ntui et al., 2010; Hao et al., 2010]. Moreover, Super-*Agrobacterium* ver.1 showed stronger inhibition of ethylene evolution and higher T-DNA transfer frequencies than chemical treatments in melon and wild water melon (Nonaka et al., 2008a; Malambane et al., 2018). For the further improvement of Super-*Agrobacterium* ver.1, a stronger promoter was used to drive *acdS*. In Super-*Agrobacterium* ver.1, the expression of *acdS* gene was under the control of the *lac* promoter, which shows constitutive expression in *A. tumefaciens*. Instead of the *lac* promoter, the *virD* promoter was cloned from *A. tumefaciens* and was used in Super-*Agrobacterium* ver. 2 (Someya et al., 2013). *virD* genes are induced by acetosyringone at pH 5.2, which is the co-cultivation condition. This promoter showed higher gene expression levels than the *lac* promoter during co-cultivation (Someya et al., 2013), resulting in higher T-DNA transfer frequencies in Super-*Agrobacterium* ver. 2 than in ver.1 (Someya et al., 2013).

Gamma-aminobutyric acid (GABA), an amino acid, was determined to be another negative factor in *Agrobacterium*-plant interactions (Chevrot et al., 2006; Haudecoeur et al., 2009; Nonaka et al., 2017). GABA is taken up into *A. tumefaciens* and triggers the degradation of the quorum-sensing (QS) signal, resulting in the reduced horizontal gene transfer of the Ti plasmid and the aggressiveness of the plant host (Chevrot et al., 2006; Haudecoeur et al., 2009). GABA is a biologically active agent in animals, plants, and bacteria. In animals, GABA is particularly well known as an effector, lowering blood pressure (Elliott and Hobbiger, 1959; Takahashi et al., 1961; Takahashi et al., 1995), and its mechanism of action has been well studied. It was found that some chemical compounds control GABA effect in animals. While contrarily, in plants, GABA was know as modulator cell elongation, abiotic stress and pathogen attack (Park et al., 2010; Renault et al., 2011; Shelp et al., 2012; Forlani et al., 2014), but the action mechanisms of GABA in-plants are still to be clarified, and the chemical compounds related with GABA perception or signal transduction in plants have not been identified. Some bacteria are known to harbor GABA transaminase (*gabT*), a GABA degradation enzyme. Utilization of GabT activities increased the transient and stable transformation frequencies in tomato and grass plants [Nonaka et al., 2017 (Super-*Agrobacterium* ver.3)]. Super-*Agrobacterium* ver. 3 was also effective in the agro-infiltration method (Hoshikawa et al., 2019; Knoch et al., 2019).

Stable transformation techniques are important as they are used for breeding GM crops. Transient transformations are also widely used in plant science; for example, protein production by excessive gene overexpression and gene function analysis by the virus-induced gene silencing (VIGS) system (Velásquez et al., 2009), are based on transient gene transfers. However, some plants have significantly lower transient gene transfer rates, creating a limitation in plant science research that should be resolved by increasing the transient transformation (T-DNA transfer) frequency in a wide variety of plant hosts. Therefore, the host range of *A. tumefaciens* must be enlarged, and its transformation efficiency increased. In this study, to further increase the transient and/or stable transformation frequencies, Super-*Agrobacterium* was updated to ver. 4 by introducing both AcdS and GabT activity to the GV2260 strain, which has similar abilities, compared with other strains such as EHA105, EHA101, LBA4404, and MP90 (Sun et al., 2006 and Chetty et al., 2013). The abilities of the Super-*Agrobacterium* ver. 4 were evaluated in *Erianthus ravennae*, *Solanum lycopersicum* “Micro-Tom,” *Nicotiana benthamiana*, and *S. torvum* for both transient and stable transformations.

## Materials and Methods

### Bacterial Strains, Vectors, and Culture Conditions

All bacterial strains and vectors, which were used in this study, were listed up in **Table 1**. The vector maps were described in **Supplemental Figure 1**. *A. tumefaciens* strains were grown at 28°C in Luria Broth (LB) medium (1% bacto-tryptone, 0.5% yeast extract, and 0.5% NaCl). Antibiotics were added at the following final concentrations: ampicillin at 100 µg/ml, gentamicin at 50 µg/ml, spectinomycin at 50 µg/ml, and kanamycin at 50 µg/ml. *A. tumefaciens* strains were then cultured on solid LB medium at 28°C for 2 days. A single colony was picked and cultured in 2 ml of LB medium at 28°C and 200 rpm for 2 days until the pre-culture reached the stationary phase. From this, 15 µl of culture was harvested and added to 15 ml of LB medium and cultured at 28°C and 200 rpm. When the O.D._600_ of the culture reached 0.7 to 0.9, the cells were then centrifuged, collected, and checked for enzymatic activity. For transformations, the pelleted bacterial cells were resuspended in liquid Murashige and Skoog (1962) (MS) containing 30 g/l glucose, and 500 µM acetosyringone at pH 5.2. The cell density was then adjusted to 0.4–0.5 at O.D._600_.

**Table 1:** List of *A. tumefaciens* strains and plasmids that are used in this study.

	Description	Reference
Strain name		
GV2260	Non-oncogenic *A. tumefaciens* strain	Deblaere et al. (1985)
C	*A. tumefaciens* GV2260 (pBBR1MCS-5)	Nonaka et al. (2008a)
V1	*A. tumefaciens* GV2260 (pBBRacdS) (Super-*Agrobacterium* ver. 1)	Nonaka et al. (2008a)
V3	*A. tumefaciens* GV2260 (pBBRgabT) (Super-*Agrobacterium* ver. 3)	Nonaka et al. (2017)
V4	*A. tumefaciens* GV2260 (pBBRacdSgabT) (Super-*Agrobacterium* ver. 4)	This study.
C-E	*A. tumefaciens* GV2260 (pBBR1MCS-5, pEKH_2_)	Nonaka et al. (2008a)
V1-E	*A. tumefaciens* GV2260 (pBBRacdS, pEKH_2_) (Super-*Agrobacterium* ver. 1)	Nonaka et al. (2008a)
V3-E	*A. tumefaciens* GV2260 (pBBRgabT, pEKH_2_) (Super-*Agrobacterium* ver. 3)	Nonaka et al. (2017)
V4-E	*A. tumefaciens* GV2260 (pBBRacdSgabT, pEKH_2_) (Super-*Agrobacterium* ver. 4)	This study.
C-G	*A. tumefaciens* GV2260 (pBBR1MCS-5, pIG121-Hm)	Nonaka et al. (2008a)
V1-G	*A. tumefaciens* GV2260 (pBBRacdS, pIG121-Hm) (Super-*Agrobacterium* ver. 1)	Nonaka et al. (2008a)
V3-G	*A. tumefaciens* GV2260 (pBBRgabT, pIG121-Hm) (Super-*Agrobacterium* ver. 3)	Nonaka et al. (2017)
V4-G	*A. tumefaciens* GV2260 (pBBRacdSgabT, pIG121-Hm) (Super-*Agrobacterium* ver. 4).	This study.
C-Q	*A. tumefaciens* GV2260 (pBBR1MCS-5, pEAQ-GFP-HT)	Nonaka et al. (2008a)
V1-Q	*A. tumefaciens* GV2260 (pBBRacdS, pEAQ-GFP-HT) (Super-*Agrobacterium* ver. 1)	Nonaka et al. (2008a)
V3-Q	*A. tumefaciens* GV2260 (pBBRgabT, pEAQ-GFP-HT) (Super-*Agrobacterium* ver. 3),	Nonaka et al. (2017)
V4-Q	*A. tumefaciens* GV2260 (pBBRacdSgabT, pEAQ-GFP-HT) (Super-*Agrobacterium* ver. 4),	This study.
Plasmid		
pBBR1MCS-5	Broad-host-range shuttle vector; Gen^R^	Kovach et al. (1995)
pBBRacdS	Overexpression vector for ACC deaminase under the control of the lac promoter; Gm^R^	Nonaka et al. (2008a)
pBBRgabT	Overexpression vector for GABA under the control of the lac promoter; Gm^R^	Nonaka et al. (2017)
pBBRacdSgabT	Overexpression vector for ACC deaminase and GABA transaminase under the control of the lac promoter; Gm^R^	This study.
pEKH_2_	pEKH2-nosPNPTII-ubiPGUS-35SPHPT, bBinary vector plasmid carrying the b-glucuronidase gene (*uidA*) between the T-borders; Sp^R^	Hoshikawa et al. (2012)
pIG121-Hm	Binary vector plasmid carrying the b-glucuronidase gene (*uidA*) between the T-borders; Km^R^	Ohta et al. (1990)
pEAQ-GFP-HT	Binary vector plasmid carrying the Green Fluorescent Protein gene (*GFP*) between the T-borders; Km^R^	Sainsbury et al. (2009)

### Construction of *acdS* and *gabT* Expression Plasmids

**Figure 1:**
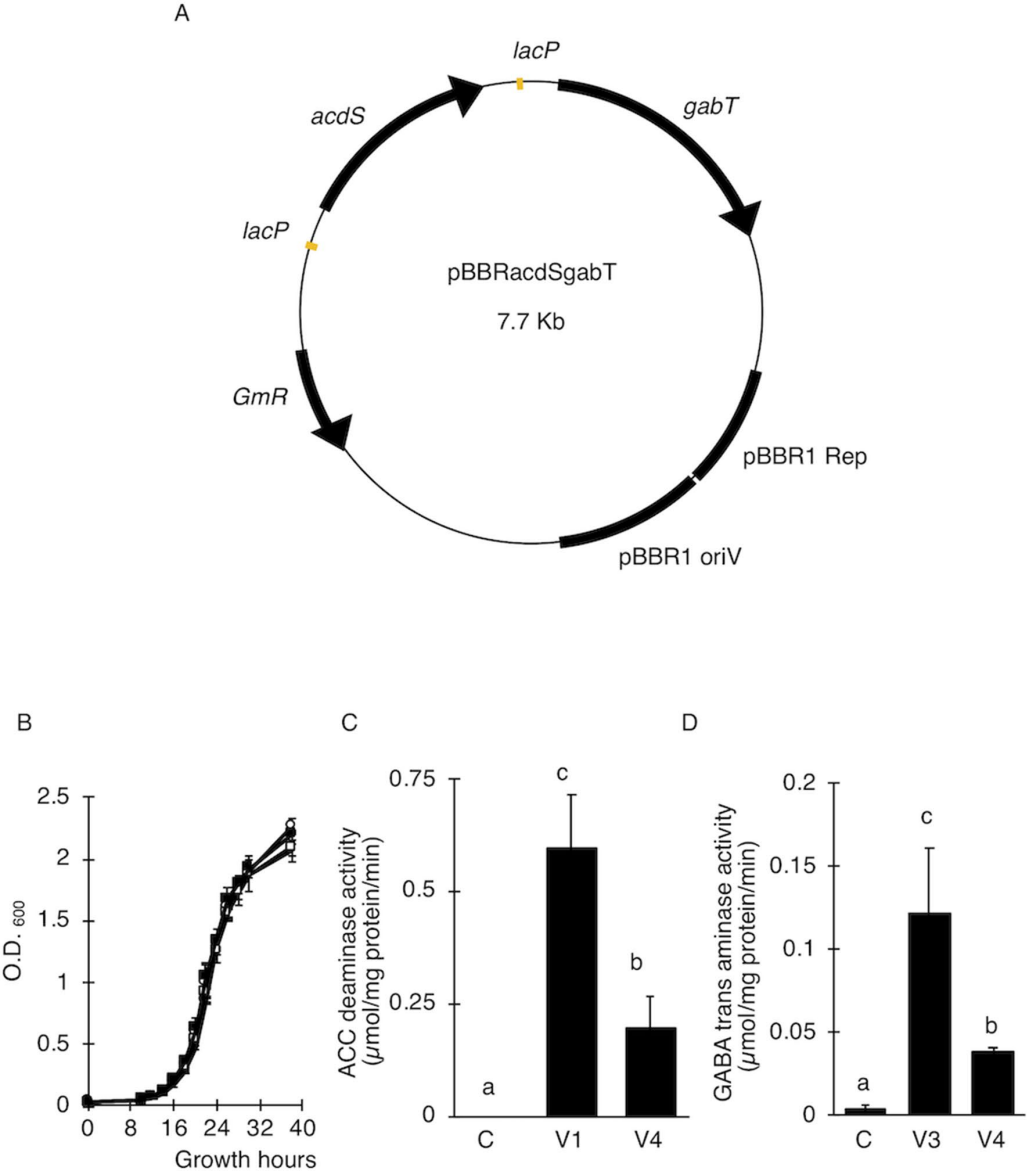
Effect of ACC deaminase and GABA transaminase activity on the transfer of T-DNA. **(A)** Map of a plasmid for the expression of ACC deaminase (*acdS*) and GABA transaminase (*gabT*) in *A. tumefaciens*. *lacP*, lac gene promoter from *E. coli*; *acdS*, ACC deaminase gene from *Psedomonas* sp (Sheehy et al., 1991, Accession No. M73488); *gabT*, GABA transaminase gene from *E. coli* (Accession No. CP040667); pBBR1 Rep, replication protein for the broad-host-range plasmid pBBR1 from *Bordetella bronchiseptica*; pBBR1 oriV, replication origin of the broad-host-range plasmid pBBR1 from *B. bronchiseptica*; pBBR1 Rep, protein for replication required by pBBR1 oriV, *GmR*, Gentamicin resistance gene. **(B)** Growth curve of *A. tumefaciens*. Open and solid circles represent *A. tumefaciens* C and V1, respectively. Open and solid squares represent V3 and V4, respectively. **(C)** ACC deaminase activity in *A. tumefaciens*. **(D)** Detection of GABA transaminase activity in *A. tumefaciens*. C, GV2260 (pBBRMCS1-5); V1, *A. tumefaciens* GV2260 (pBBRacdS); V3, *A. tumefaciens* GV2260 (pBBRgabT); V4, *A. tumefaciens* GV2260 (pBBRacdSgabT). Bars represent the standard deviation (n = 3). Different characters indicate values that were statistically different in the one-way ANOVA and Tukey-Kramer method, multiple comparison method (*P* < 0.01).

The *gabT* gene was cloned from pBBRgabT in a previous study (Nonaka et al., 2017) by polymerase chain reaction (PCR) with the primers acdSF (5′- TCTGCGCGTAATCTGCTGCTTGAGCGCAACGCAATTAATG -3′) and gabTR (5′- CGATTCTGGACTACTGCTTCGCCTCATCAAAAC-3′). The transcription terminal sequence of the ampicillin resistance gene was cloned from the pUC18 vector using PCR with the primer’s amp_ter-for2 (5′-GCTAGAATTCCTGTCAGACCAAGTTTACTC-3′) and amp_ter-rev2 (5′-CATTAATTGCGTTGCGCTCAAGCAGCAGATTACGCGCAGA-3′). Then, the two fragments were combined by fusion-PCR with the primer’s amp_ter-for2 and gabTR. The ligated fragment was inserted into pBBRacdS (Nonaka et al., 2008a) and digested with *Eco*RI and *Xba*I (New England Biolabs, Hirchin, UK). The expression of both genes was under the control of the *lacZ* gene promoters (pBBRacdSgabT, **Figure 1A**). pBBR1MCS5, pBBRacdS, pBBRgabT, and pBBRacdSgabT were introduced into *A. tumefaciens* GV2260 (pEKH_2_-nosPNPTII-ubiPGUS-35SPHPT; pEKH_2_), *A. tumefaciens* GV2260 (pIG121-Hm), or *A. tumefaciens* GV2260 (pEAQ-GFP-HT) *via* electroporation.

### ACC Deaminase Activity Assay

Cells were collected and washed twice with 100 mM Tris–HCl (pH 8.5) and resuspended in 1.5 ml of lysate buffer. The cells were lysed on ice by sonication and centrifuged at 5,000 × *g* at 4ºC for 15 min. The AcdS activity was measured according to a modified protocol based on that of Honma and Shimomura (1978). The AcdS activity was measured spectrophotometrically at 340 nm. The protein content of the extracts was determined using the Bradford method (Bradford, 1976).

### GABA Transaminase Activity in *A. tumefaciens*

The pellet of *A. tumefaciens* was re-suspended in 100 µl of BugBuster Master mix (Novagen, MA, USA) for lysate preparation. The protein concentration of the lysate was measured with a BCA Protein Assay Kit (Novagen, MA, USA). The amount of protein was adjusted to 100 µg *per* reaction mixture. The reaction mixture contained 0.1 M bicine–NaOH, 0.1 M pyridoxal phosphate, 10 mM 2-ketoglutarate, 10 mM GABA, and a proteinase inhibitor cocktail. The reaction mixture was incubated at 37°C. GabT metabolizes GABA to glutamate; therefore, to estimate the GabT activity, we detected the glutamate concentration in the reaction mixture using a Yamaki glutamate assay kit (Yamaki, Tokyo, Japan) (Akihiro et al., 2008).

### T-DNA Transfer Assay in *E. ravennae* and *S. lycopersicum*

Calli of *E. ravennae* were kindly provided by Prof. Masahiro Mii from Chiba University, Japan. The calli were induced directly from the seeds on MS medium, containing 1 g/l casamino acids, 2 mg/l 2,4-dichlorophenoxyacetic acid (2,4-D), 0.2 mg/l 6-benzylaminopurine (BAP), 30 g/l 4-O-α-D-glycopyranosyl-D-glycopyranose (maltose H) (Wako, Tokyo, Japan), and 3% Gelrite (Wako, Tokyo, Japan), were subcultured for 2 weeks before inoculation. After co-cultivation, the β-glucuronidase (GUS) activity of the *E. ravennae* calli were assayed histochemical with X-Gluc buffer containing 100 mM phosphate buffer, 10 mM EDTA, 2.5 mM potassium ferricyanide, 2.5 mM potassium ferrocyanide, 0.1% Triton X-100, and 0.5 mg/l X-glucuronide. The GUS-stained calli were observed using a stereoscopic microscope (Leica: MX FLIII, DFC300 FX, Application Suite, Leica, Germany), the number of GUS-stained spots per 1 g of calli calculated, and the T-DNA transfer efficiency was estimated, based on the relative number of GUS spots.

Tomato seeds were washed with 70% ethanol for 10 s, sterilized with 5% hypochlorous acid containing 10% Triton X-100 for 45 min, and washed three times with sterilized water. After the third wash, the seeds were kept in water for 2 days. The sterilized tomato seeds were sown on MS medium, containing 15 g/L sucrose (Wako, Tokyo, Japan) and 0.3% Gelrite (Wako, Tokyo, Japan). Cotyledons from 7-day-old tomato seedlings were cut into four pieces and used to generate two locations for inoculations with *A. tumefaciens*. Thirty explants were subjected to each treatment. The inoculated explants were cultured on co-cultivation medium (pH 5.2) containing MS salts, 30 g/L glucose, 500 µM acetosyringone, and 0.3% Gelrite (Wako, Tokyo, Japan) at 25°C, for 3 days in the dark. After 3 days of co-cultivation, the tomato explants were assayed histochemically for GUS activity with X-Gluc buffer, described above. GUS stained tomato cotyledon explants were observed and images were taken using a stereoscopic microscope system (Leica: MX FLIII, DFC300 FX, Application Suite, Leica, Wetzlar, Germany). The GUS stained areas were converted into numerical values by Image J (National Institutes of Health: http://rsbweb.nih.gov/ij/) and the percentage of GUS stained area for each explant was calculated. According to the results, GUS stained tomato explants were categorized into 4 classes: (Class 1) less than 5%, (Class 2) 5–10%, (Class 3) 10–20%, and (Class 4) more than 20%. To estimate the T-DNA transfer, the frequency of more than 20% was calculated.

### Tomato Stable Transformation

Tomato transformations followed the protocol by Sun et al. (2006). In brief, after 3 days of co-cultivation, tomato cotyledon segments were placed on a callus-induction medium [MS medium containing 0.3% Gelrite (Wako, Tokyo, Japan), 1.5 mg/l zeatin, 100 mg/l kanamycin, and 375 mg/l Augmentin (GlaxoSmithKline, London, UK)] for 4 weeks. Calli that formed segments were cultured on shoot-induction medium [MS medium containing 0.3% Gelrite (Wako, Tokyo, Japan), 1.0 mg/l zeatin, 100 mg/l kanamycin, and 375 mg/l Augmentin (GlaxoSmithKline, London, UK)] for 4 weeks. The shoots were then placed on rooting medium, which consisted of half-strength MS medium, 0.3% Gelrite (Wako, Tokyo, Japan), 100 mg/L kanamycin, and 375 mg/l Augmentin, for 2 weeks. Tissues were each subcultured for 10–14 days.

### Agro Infiltration Method

*A. tumefaciens* GV2260 carrying pEAQ-GFP-HT (Sainsbury et al., 2009) was grown in LB media, resuspended in 10 mM MgCl_2_, 10 mM MES, pH 5.6, 150 µM acetosyringone, and incubated for 3 h at room temperature. The leaves were then syringe infiltrated with the *A. tumefaciens*. Concentrations of *A. tumefaciens* were 0.3 at O.D._600_ for *N. benthamiana* and 1 at O.D._600_ for *S. torvum*. GFP fluorescence was detected 3 and 5 days after infiltration for *N. benthamiana* and *S. torvum*, respectively. Each experiment was repeated three times.

### Ploidy Analysis

The ploidy of the rooting shoots was checked with flow cytometry. One square centimeter of leaf was cut from the rooting shoots, chopped, and added to 250 µl of nucleus-extraction solution (CyStain UV Precise P, Sysmex, Hyogo, Japan). To purify the nucleus-extraction solution, 1 mm^2^ mesh was used. After purification, 1 ml of staining solution (CyStain UV Precise P, Sysmex, Hyogo, Japan) was added and incubated for 1 min. This solution was applied to an Attune focusing analyzer (ABI, CA, USA), and 2n plants were selected. The 2n plants were then planted on solid medium and acclimatized.

### Southern Blot Analysis

Genomic DNA was extracted from young tomato leaves using Maxwell 16 System DNA Purification kits (Promega, WI, USA). The purified DNA was digested with *Hin*dIII, electrophoretically separated in 0.8% agarose gel, and transferred onto Gene Screen Plus nylon membranes (Roche Diagnostics, Basel, Swiss) with 20× saline–sodium citrate (SSC) buffer. After ultraviolet (UV) cross-linking, the membranes were hybridized in a solution containing 7% sodium dodecyl sulfate (SDS), 50% deionized formamide, 50 mM sodium phosphate (pH 7.0), 2% blocking solution, 0.1% N-lauroylsarcosine, 0.75 M NaCl, and 75 mM sodium citrate at 42ºC overnight. For hybridization, a digoxigenin (DIG)-labeled DNA probe, specific for *nptII* (0.8 Kb), was used. A DIG-labeled probe was generated by DIG-High Prime, and the DIG signal was detected according to the manufacturer’s protocol (Roche Diagnostics, Basel, Swiss).

### Statistical Analysis

The average values were obtained from three biological replicates. One-way analysis of variance (ANOVA) and Tukey Kramer’s multiple range test, with *P* < 0.01 or *P* < 0.05, were carried out to determine the significant differences. Statistical analyses were carried out using the SAS statistics program (version 8.0, SAS Institute Cary, NC, USA).

## Results

### Introduction of AcdS and GabT Activity Did Not Affect Bacterial Growth

Since ethylene and GABA suppress the transfer of T-DNA in different ways, we predicted that the introduction of AcdS and GabT activity into *A. tumefaciens* would be effective at increasing the T-DNA transfer. These two genes were introduced by pBBR1MCS-5, the broad host range plasmid (Kovach et al., 1995) (**Figure 1A**, pBBRacdSgabT), and expressed under the control of the *lac* promoter (Nonaka et al., 2008a; Nonaka et al., 2017). To estimate whether these two genes affect bacterial growth or not, growth curves were compared for the four strains [(C), (V1), (V3), and (V4)]. In all strains, the accelerated growth period began 10 h after culturing, and after 18 to 26 h, the logarithmic growth phases were observed (**Figure 1B**). These results indicate that introducing the *acdS* and *gabT* at the same time in *A. tumefaciens* did not affect its bacterial growth. To measure the AcdS and GabT activity, cells were collected by centrifugation, and the lysate was prepared. Then, the AcdS and GabT activity were measured, as described in previous studies (Nonaka et al., 2008a; Nonaka et al., 2017). Both activities were detected in the V4 strain, but the AcdS and GabT activities in V4 were one-third of the V1 and V3, respectively (**Figures 1C, D**).

### Evaluation of the Super-*Agrobacterium* for T-DNA Transfer in Plants

**Figure 2:**
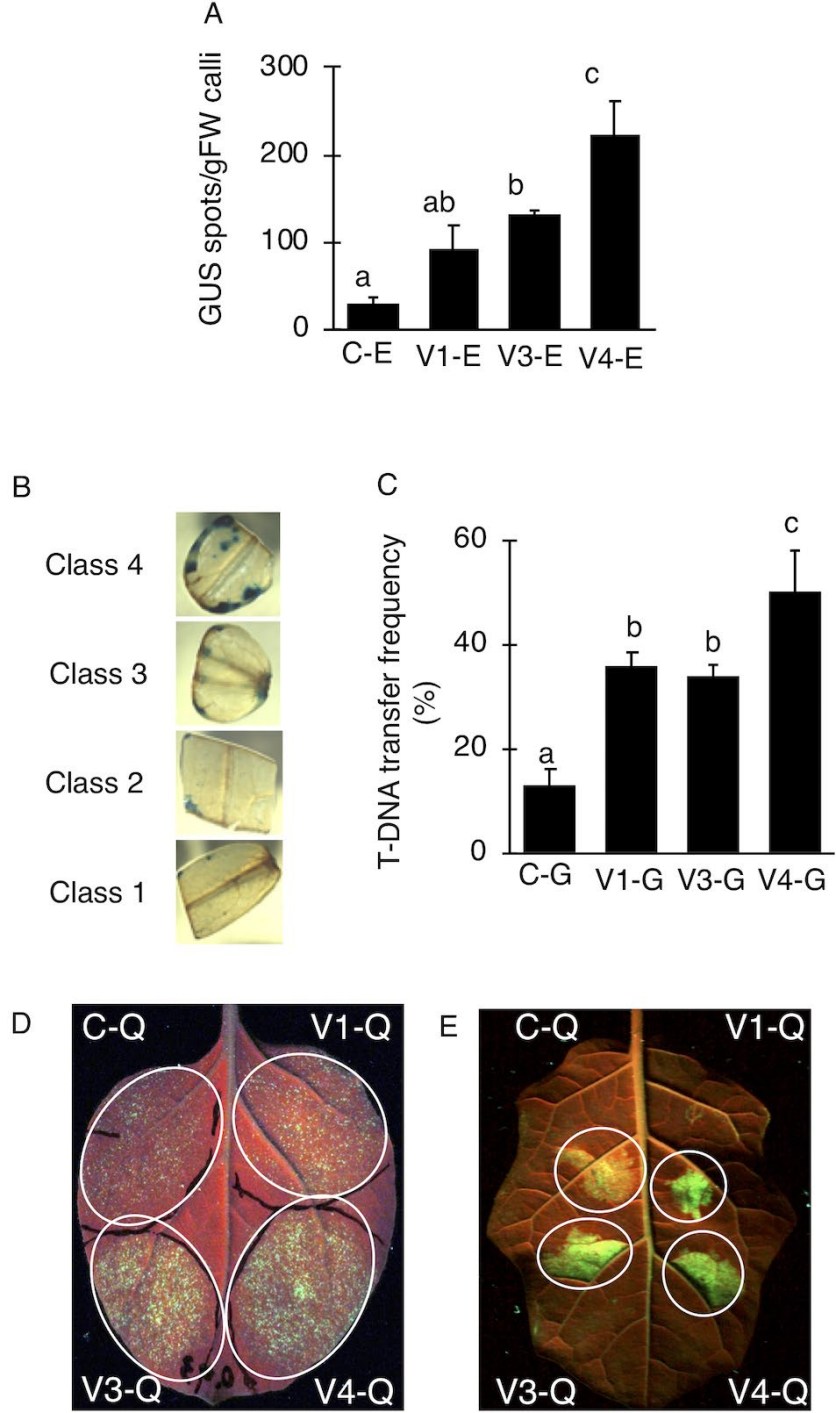
Transient transformations in tomato *via* tissue culture and co-cultivation method. **(A)** Occurrence of T-DNA transformations in *E. ravennae*. The number of GUS-stained spots *per* 1 g of *E. ravennae* calli were counted for each treatment. The bars indicate the standard deviation (n = 3). Different characters indicate values that were significantly different according to the one-way analysis of variance, multiple comparison method (*P* < 0.01). C-E: *A. tumefaciens* GV2260 (pBBR1MCS-5, pEKH_2_), V1-E: *A. tumefaciens* GV2260 (pBBRacdS. pEKH_2_), V3-E: *A. tumefaciens* GV2260 (pBBRgabT, pEKH_2_), V4-E: *A. tumefaciens* GV2260 (pBBRacdSgabT, pEKH_2_). **(B)** GUS stained explants of *S. lycopersicum* “Micro-Tom.” Explants were prepared from 7 days old seedlings. After 3 days of co-cultivation, explants were stained. Classification of GUS-stained cotyledon explants. GUS stained tomato cotyledons were categorized depending on the stained area. Categorized into 4 classes: (Class 1) less than 5%, (Class 2) 5–10%, (Class 3) 10–20%, and (Class 4) more than 20%. **(C)** Appearance ratio Class 4 in tomato cotyledons. C-G: *A. tumefaciens* GV2260 (pBBRMCS1-5, pIG121-Hm); V1-G: *A. tumefaciens* GV2260 (pBBRacdS, pIG121-Hm); V3-G: *A. tumefaciens* GV2260 (pBBRgabT, pIG121-Hm); V4-G: *A. tumefaciens* GV2260 (pBBRacdSgabT, pIG121-Hm). Bars represent the standard deviation (n = 3). Different characters indicate values that were statistically different in a one-way ANOVA and the Tukey-Kramer method, multiple comparison method (*P* < 0.01). **(D)** Transient transformation *via* agroinfiltration methods on *N. benthamiana*. **(E)** Transient transformation *via* Agroinfiltration methods on *S. torvum*. GFP signals were used as indicators of transformation. C-Q: *A. tumefaciens* GV2260 (pBBRMCS1-5, pEAQ-GFP-HT); V1-Q: *A. tumefaciens* GV2260 (pBBRacdS, pEAQ-GFP-HT); V3-Q: *A. tumefaciens* GV2260 (pBBRgabT, pEAQ-GFP-HT); V4-Q: *A. tumefaciens* GV2260 (pBBRacdSgabT, pEAQ-GFP-HT).

To examine whether the AcdS and GabT activities were enough to increase the transfer of T-DNA, the T-DNA transfers in *E. ravennae* and *S. lycopersicum* “Micro-Tom” were observed. *E. ravennae* is known for its high bio-mass production and is relevant for practical agriculture. After 3 days of co-cultivation, the number of blue spots were counted to evaluate the T-DNA transfer in *E. ravennae*. Four strains, (C-E), (V1-E), (V3-E), and (V4-E) were used for the transformation. The V1-E, V3-E, and V4-E showed higher T-DNA transfer frequencies than the control, C-E. The inoculation of V4-E increased the T-DNA transfer frequency by 7.2, 2.4, and 1.7 times, compared to the C-E, V1-E, and V3-E, respectively (**Figure 2A**). Next, we evaluated the abilities of Super-*Agrobacterium* V4 using *S. lycopersicum* “Micro-Tom.” Almost 100 explants of “Micro-Tom” were inoculated for each bacterial strain [(C-G), (V1-G), (V3-G), and (V4-G)]. The *uidA* gene was used as an indicator of T-DNA transfer, and the blue area indicated transformed cells (**Figure 2B**). The GUS-stained area was determined in each of the explants with Image J, as described in the *Materials and Methods* section (in “2.5 T-DNA transfer assay in *E. ravennae* and *S. lycopersicum*”). The degree of staining was categorized into 4 classes (**Figure 2B**). To evaluate the ability of the T-DNA transfer in C-G, V1-G, V3-G, and V4-G, the frequency of class 4 was compared. V4-G showed the highest frequency of class 4; the frequencies were 3.9, 1.4, and 1.5 times higher than the C-G, V1-G, and V3-G, respectively. V1-G and V3-G showed almost the same levels (**Figure 2C**). Therefore, the activities of AcdS and GabT in V4-E and V4-G, were enough to increase the T-DNA transfer frequencies in *E. ravennae* and *S. lycopersicum* “Micro-Tom.” Additionally, we evaluated the ability of T-DNA transformation with V4 in the Agroinfiltration method. Plasmid pEAQ-GFP-HT was used as a binary vector in Agroinfiltration method. *N. benthamiana* and *S. torvum* were inoculated with four strains [(C-Q), (V1-Q), (V3-Q), and (V4-Q)]. The strength of the GFP signal was used as an indicator of the frequency of the T-DNA transfer. In *N. benthamiana*, V4-Q induced higher GFP expression than the C-Q strain and V1-Q (**Figure 2D**), but it was the same level as the V3-Q. In *S. torvum*, the success of the V4-Q strain with the Agroinfiltration treatment was greater than that of the C-Q strain, but the same as that of the V1-Q and V3-Q strains (**Figure 2E**).

### *A. tumefaciens* With Both AcdS and GabT Activities Resulted in the Enhanced Stable Transformation of Tomato

**Figure 3:**
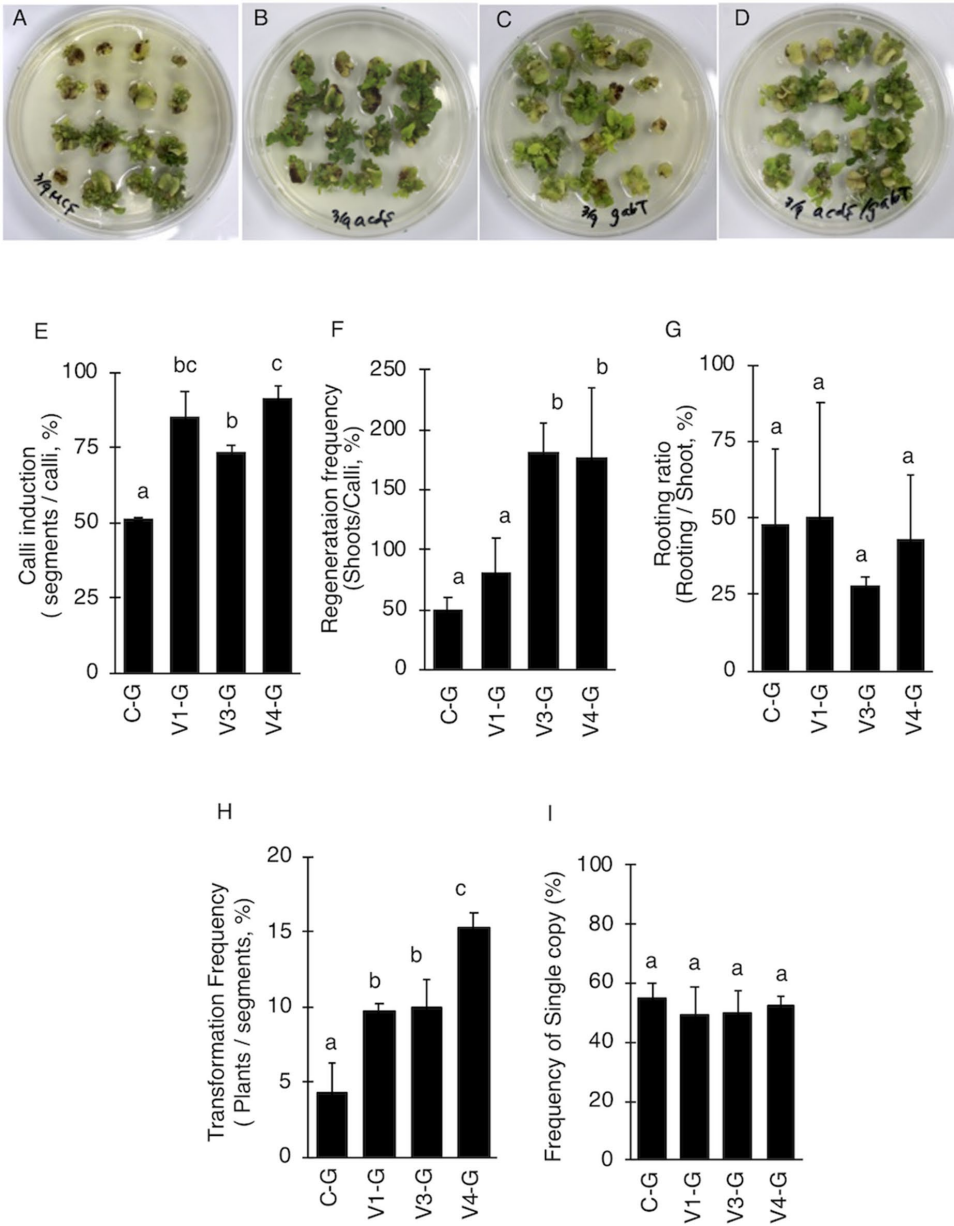
Effect of AcdS and GabT activity on regeneration and stable transformation. Regenerated shoots from “Micro-Tom” calli inoculated with **(A)** C-G, **(B)** V1-G, **(C)** V3-G, and **(D)** V4-G. **(E)** Appearance of calli, **(F)** Frequency of regeneration, **(G)** Rooting ratio, **(H)** Frequency of calli regeneration. **(I)** Frequency of appearence for transgenic tomato plants which have single copy of T-DNA. C-G: *A. tumefaciens* GV2260 (pBBRMCS1-5, pIG121-Hm); V1-G: *A. tumefaciens* GV2260 (pBBRacdS, pIG121-Hm); V3-G: *A. tumefaciens* GV2260 (pBBRgabT, pIG121-Hm); V4-G: *A. tumefaciens* GV2260 (pBBRacdSgabT, pIG121-Hm). Bars represent the standard deviation (n = 3). Different characters indicate statistical differences in a one-way ANOVA and the Tukey-Kramer method, multiple comparison method (*P* < 0.01).

V4 was effective at the T-DNA transfer in *E. ravennae* and *S. lycopersicum* “Micro-Tom” (**Figures 2A, C**), however this is one step of the stable transformation process. The entire process for *Agrobacterium*-mediated stable transformation is divided into four steps: i) T-DNA transfer and integration into the plant genome, ii) calli induction, iii) the regeneration of the shoots, and iv) rooting. It was not clear if the V4 affected these other steps. To ascertain this information, the frequency of each process was observed in *S. lycopersicum* “Micro-Tom,” which has a well-established regeneration system for processes (ii) to (iv) (Sun et al., 2006). To characterize each strain, C-G, V1-G, V3-G, and V4-G were used. One-month after inoculation, the calli inductions were observed. All Super-*Agrobacterium* strains increased the callus inductions compared with the C-G (**Figures 3A–D**). V1-G and V4-G showed slightly higher calli induction ratios (calli number / segments number) than the V3-G. The C-G, V1-G, V3-G, and V4-G showed calli induction frequencies of 51.5 ± 0.6, 85.2 ± 8.8, 73.8 ± 2.03, and 91.8 ± 3.7%, respectively (**Figure 3E**, **Table 2**). Shoot regeneration ratios (shooting number / calli number) were increased with the inoculation of the V3-G and V4-G. V1-G was slightly higher than that of the C-G. The frequency of the shoot regeneration ratios with the inoculations of C-G, V1-G, V3-G, and V4-G were 49.7 ± 10.9, 80.4 ± 29.2, 181.8 ± 23.4, and 176.3 ± 58.9%, respectively (**Figure 3F**, **Table 2**). The frequencies for rooting from the shoots (rooting number/ shoots number) were similar for all strains (**Figure 3G**, **Table 2**). These results suggest that V1-G had positive effects on step (ii) calli induction, V3-G increased step (iii) shooting, and V4-G accelerated both steps (ii) and (iii) in the *Agrobacterium*-mediated stable transformation process. After regenerated diploid shoots (2n) were selected, the exogenous T-DNA was detected by PCR (data not shown) and Southern hybridization analysis (**Supplemental Figure 2**). The stable transformation frequencies were evaluated with single-copy-number plants, and identification by Southern hybridization analysis. These results imply that all of the lines we obtained were independent and did not contain a cloned plant. The C-G, V1-G, V3-G, and V4-G showed the stable transformation efficiencies of 4.3 ± 1.9, 9.7 ± 0.4, 9.8 ± 1.6, and 15.2 ± 1.1%, respectively (mean ± SD of three repetitions) (**Figure 3H** and **Table 2**). Thus, V4 exhibited approximately 3.6, 1.6, and 1.6 times the stable transformation frequency of C-G, V1-G, and V3-G, respectively. The frequency of regenerated rooting shoot with single copy was same level in all *A. tumefaciens* strains (**Figure 3I**).

**Table 2:** Effect of the Super-Agrobacterium ver.1, ver.3, and ver.4 on plant regeneration and transformation of the ‘Micro-Tom’ cotyledons.

		Numbers of						Transformation frequency
Experiment repetition	Agrobacterium strain	Segments	Calli	Shoots	Rooting	Diploid	Single copy	Inoculated Segments
								/Single copy (%)
1st	C-G	110	56	26	7	5	3	2.7
	V1-G	125	94	52	47	19	12	9.6
	V3-G	92	80	152	39	14	8	8.7
	V4-G	96	84	163	59	29	14	14.6
2nd	C-G	94	49	20	15	11	6	6.4
	V1-G	88	78	88	15	23	9	10.2
	V3-G	95	72	144	40	22	11	11.6
	V4-G	75	70	157	41	20	11	14. 7
3rd	C-G	82	42	26	11	6	3	3.7
	V1-G	85	78	57	25	16	8	9.4
	V3-G	88	65	101	31	16	8	9.1
	V4-G	91	86	95	63	28	15	16.5

Each column “Segments,” “Calli,” “Shoots,” “Rooting,” “Diploid,” and “Single copy” indicated the number of occurrences. “Segments” means the number of segments used for inoculation of A. tumefaciens. “Calli,” “Shoots,” and “Rooting” showed the number of regenerate calli, shoot, and rooting. “Diploid” was the number rooting shoots that were diploid, detected by the ploidy analyzer. “Single copy” means the number of diploid rooting shoots with single copy of T-DNA, identified by Southern blot analysis. Transformation frequency was calculated as follows: the total number of transgenic plants with diploid and single copy was divided by the number of explants inoculated and then multiplied by 100. Only one plant regenerated per cotyledon explant was considered to calculate transformation efficiency. Three replications were done for this experiment.

## Discussion

*A. tumefaciens* with AcdS and GabT was expected to cause reduced ethylene and GABA content in plants. Indeed, the ethylene levels in the plant tissues during the transformation were reduced by the *A. tumefaciens* with AcdS activity (Nonaka et al., 2008a; Malambane et al., 2018). On the other hand, significant differences in GABA content during the co-cultivation were not observed between *A. tumefaciens* with GabT activity and the control (data not shown). As *A. tumefaciens* takes the GABA from the plant into the bacterial cell through a kind of ABC transporter (Planamente et al., 2010; Planamente et al., 2013), if the GabT activity introduced *A. tumefaciens*, GABA taken into bacterial cell would be degraded. The degradation of GABA occurred only in bacterial cells, and at very localized areas. Therefore, it was difficult to detect the differences of GABA content. ACC deaminase activity and GabT activity in *A. tumefaciens* were effective at increasing the T-DNA transfer frequency (Nonaka et al., 2008a; Nonaka et al., 2017; Ntui et al., 2010; Hao et al., 2010), but it was not clear which was more effective in *Agrobacterium*-plant interactions. To ascertain this, the T-DNA transfer abilities of Super-*Agrobacterium* ver. 1 and ver. 3 were compared in *E. ravennae* and tomato with the tissue culture and co-cultivation methods. No differences were observed between the strains in *E. ravennae* and *S. lycopersicum* “Micro-Tom” (**Figures 2A, C**). With the Agroinfiltration method, the same tendency was observed in *S. torvum* (**Figure 2E**). These results mean that ethylene and GABA influence the T-DNA transfer frequencies at almost the same level in these plant species. On the other hand, in *N. benthamiana*, Super-*Agrobacterium* ver. 3 and ver. 4 showed higher level of T-DNA transfer than GV2260 and Super-*Agrobacterium* ver. 1, but the level of T-DNA transfer was same in ver. 3 and ver. 4. This showed that in the *N. benthaminana*, AcdS activity did not improve the T-DNA transfer, but GabT activity was effective at increasing the T-DNA transfer. Therefore, in *N. benthamiana*, GABA is a stronger negative factor than ethylene. From these results, the effect of the Super-*Agrobacterium* was found to be different, dependent on the plant species, thus the selection of the most suitable strain is important for the successful application of the technology.

Even under conditions where the *vir* gene is sufficiently expressed, our Super-*Agrobacterium* strains could further improve T-DNA transfer. In this study, with the tissue culture and co-cultivation methods, 500 µM of acetosyringone, which was enough to induce *vir* gene expression (Nonaka et al., 2008b), was used during co-cultivation. Super-*Agrobacterium* ver. 1, ver. 3, and ver. 4 further increased the T-DNA transfer frequency, despite the existence of enough *vir* gene inducers (**Figures 2A, C**). The additional effects of AcdS and GabT under the acetosyringone indicate that in the T-DNA transfer, the ethylene and GABA affect different from the level of *vir* gene inducer. Previous studies have demonstrated that GABA was independent of *vir* gene expression (Chevrot et al., 2006; Haudecoeur et al., 2009). Thus, the inhibition of GABA further increased the transformation. On the other hand, research has shown that the ethylene target points are involved with *vir* gene expression, and the ethylene perceiving plant would reduce *vir* gene inducers or release antagonists of the *vir* gene inducers (Nonaka et al., 2008b). If the target point of the ethylene was the reduction of the *vir* gene inducer, the effect of the Super-*Agrobacterium* ver. 1 would be masked by acetosyringone. In fact, our results showed that the Super-*Agrobacterium* ver. 1 increased the transformation frequency up to 3.2 and 2.8 times in *E. ravennae* and *S. lycopersicum*, respectively, even with the application of the *vir* gene inducers. Therefore, these results suggest that the target point of ethylene is not the reduction of *vir* gene inducers, but the suppression of the antagonists.

In Super-*Agrobacterium* ver. 4, the enzymatic activity was one third of the Super-*Agrobacterium* ver. 1 and ver. 3, but it was effective in *E. ravennae* and *S. lycopersicum* “Micro-Tom” (**Figures 2A, C**). The amount of AcdS protein in the Super-*Agrobacterium* ver. 4 was one third of that found in Super-*Agrobacterium* ver. 1 (**Supplemental Figure 3**). Expressing multiple genes using the same promoter may reduce the expression levels of each gene. In this study, we used the lac promoter to drive both the *acdS* and *gabT* genes. If stronger promoters were used, the expression levels would be increased. Previous research compared the *vir* gene promoters (*virB*, *virC*, and *virD*) with the *lac* promoter activities; the *vir* gene promoters were found to show higher promoter activities than the *lac* promoter (Someya et al., 2013). Therefore, using these promoters would be effective to increase *acdS* and *gabT* expression in the Super-*Agrobacterium* ver. 4. Indeed, replacement of the promoter increased the *acdS* gene expression and the activity in *A. tumefaciens*, resulting in increased T-DNA transformation frequencies (Super-*Agrobacterium* ver. 2) (Someya et al., 2013). Therefore, replacing promoters would increase the transient transformations in *S. torvumvia* the agroinfiltration method.

In this study, the stable transformation frequency was 15.2 ± 1.1%. This value was calculated as the ratio between independently transformed plants with diploid and single copy number in soil (checked by a ploidy analyzer and Southern blot analysis) and the total number of explants infected with *A. tumefaciens*. Previous studies have reported transformation frequencies that differ from ours (Sun et al., 2006; Khoudi et al., 2009; Khuong et al., 2013; Chetty et al., 2013). The transformation frequency might depend on the bacterial strain, binary vector and the selection method. In our study, the transformation frequency was calculated using regenerated rooting shoot with diploid and a single copy *per* inoculated segment. In contrast, most previous studies calculated this frequency from the PCR-positive tomatoes (Sun et al., 2006; Khoudi et al., 2009; Khuong et al., 2013; Chetty et al., 2013). Therefore, it would be difficult to compare between our and previous results. To create a transgenic plant, it is important to avoid somaclonal variation and multiple copies, which our method did.

The process of *Agrobacterium*-mediated stable transformation contains four steps: i) the T-DNA transfer into plant cells and integration into the host genome, ii) callus induction, iii) the regeneration of shoots, and iv) rooting. The activity of AcdS and GabT increased step (i) (**Figures 2A, C**). In the rooting step, there were no significant differences detected between them. Other steps showed different responses to AcdS and/or GabT activity (**Figure 3** and **Table 2**). The AcdS activity showed higher callus induction frequencies than the GabT activity, whereas the GabT activity induced higher shoot regeneration ratios than the AcdS. The browning callus appearance was suppressed by *A. tumefaciens* with AcdS (**Figures 3A–D**), as the ethylene induced hypersensitive responses and programmed cell death (Wang et al., 2017); infection of *A. tumefaciens* with AcdS (Super-*Agrobacterium* ver. 1 or ver. 4) with the ability to remove ethylene, suppressed the browning phenomena. Although the function of GABA in plants needs to be clarified, there have been several studies regarding the functions of GABA as a signaling compound in plant growth and development. The increased endogenous concentrations of GABA seem to be the reason for impaired cell elongation in the *Arabidopsis thaliana* mutants, *pop2*, and *her1*, and the corresponding phenotypes (Renault et al., 2011). Infection of *A. tumefaciens* with GabT activity locally decreased GABA content in the plant calli and maintained higher shoot regeneration frequencies. Since both activities have different effective points in the transformation process, Super-*Agrobacterium* ver. 4. with AcdS and GabT activity at the same time, enhanced the stable transformation frequency approximately 3.6 times, compared with that of the original GV2260 strain.

We succeeded in producing an *A. tumefaciens* strain with improved potential for transformation by imbuing it with the ability to remove ethylene and GABA, which are negative factors in the *Agrobacterium*-plant interactions. *A. tumefaciens* with AcdS and GabT increased the T-DNA tranfer and stable transformation frequency. Especially in tomato, this newly bred bacterium (Super-*Agrobacterium* ver. 4) enables us to decrease the number of cotyledons used for transformation and allows us to reduce 72% of the time and labor required for transformation. Moreover, because our system was the plasmid with *acdS* and *gabT* gene, it is used in combination with other strains, such as the EHA105, EHA101, LBA4404, MP90, and AGL1. Based on this, we conclude that this new system is a useful tool for plant genetic engineering. On the other hand, the frequency is still not enough depending on the plant species and cultivars (**Figures 3E, F**). Therefore, the additional effort should have been required to adapt *Agrobacterium*-mediated transformation for a wide variety of plants. Other negative factors in *Agrobacterium*-plant interactions, aside from ethylene and GABA, have been reported by previous studies (Liu and Nester, 2006; Yuan et al., 2007; Yuan et al., 2008; Anand et al., 2008). Therefore, to expand plant spices and cultivars adapting *Agrobacterium*-mediated transformation, multiply suppress of these negative factors would be also effective.

## Data Availability Statement

All datasets GENERATED for this study are included in the manuscript/**Supplementary Files**.

## Author Contributions

SN designed the experiments, analyzed the data, and wrote the manuscript. TS constructed the plasmid pBBRacdSgabT and did the western blot analysis. YK performed the experiments about Agroinfiltration. HE and KN critically revised and approved the manuscript for publication.

## Funding

This research was supported by grants from the New Energy and Industrial Technology Development Organization (NEDO) to HE and from JSPS KAKENHI (Grant Numbers JP24780001 and JP19K05964) to SN. Cooperative Research Grant from Plant Transgenic Design Initiative (PTraD), Gene Research Center in Tsukuba Innovation Plant Research Center (T-PIRC), University of Tsukuba, Japan supported this research.

## Conflict of Interest

The authors declare that the research was conducted in the absence of any commercial or financial relationships that could be construed as a potential conflict of interest.

## Supplementary Material

Supplemental Figure 1 Map of binary vectors. **(A)** Map of pEKH_2_-nosPNPTII-ubiPGUS-35SPHPT. NosP; Nopalin synthesis gene promoter, UbiP; ubiquitin gene promoter from rice, NosT; Nopalin synthesis gene terminator, *nptII*; neomycin phosphotransferase gene, *uidA*; beta-glucuronidase gene, *hptII*; hygromycin phosphotransferase gene, OriV; replication origin V (IncPα, plasmid RK2 from *E. coli*, GeneBank accession #J01780), *KanR*; Kanamycin resistance gene. RB; Right border sequence, LB; Left border sequence. **(B)** Map of pIG121-Hm. NosP; Nopalin synthesis gene promoter, CaMV 35S P; Cauliflower mosaic virus 35S promoter, NosT; Nopalin synthesis gene terminator, *nptII*; neomycin phosphotransferase gene, *uidA*; beta-glucuronidase gene, *hptII*; hygromycin phosphotransferase gene, OriV; replication origin V (IncPα, plasmid RK2 from *E. coli*, GeneBank accession #J01780), *KanR*; Kanamycin resistance gene, RB; Right border sequence, LB; Left border sequence. **(C)** Map of pEAQ-GFP-HT. CaMV 35S P; Cauliflower mosaic virus 35S promoter, NosT; Nopalin synthesis gene terminator, GFP; green fluorescence gene, RB; Right border sequence, LB; Left border sequence.

Supplemental Figure 2 Map of the pIG121-Hm vector and the southern blot analysis of the T_0_ generation. **(A)** Present the maps of the T-DNA region in the pIG121-Hm expression vectors used for the stable transformation. Red bars represent the position of probes used in the southern blot analysis. *Hin*dIII indicates the restriction enzyme sites that were used in the southern hybridization. NPTII probes were used in. **(B)** Southern blot analysis of the T_0_ generation. Red numbers indicate the transgenic lines with single copy. C-G: *A. tumefaciens* GV2260 (pBBRMCS1-5, pIG121-Hm); V1-G: *A. tumefaciens* GV2260 (pBBRacdS, pIG121-Hm); V3-G: *A. tumefaciens* GV2260 (pBBRgabT, pIG121-Hm); V4-G: *A. tumefaciens* GV2260 (pBBRacdSgabT, pIG121-Hm).

Supplemental Figure 3 Western blot analysis of ACC deaminase expression in *A. tumefaciens* strains using the cell lysate at the ‘Early stage’ (O.D._600_ 0.7). ACC deaminase was probed with an anti-ACC deaminase antibody. Coomassie Brilliant Blue staining (bottom panel) is shown as an internal control. C-G: *A. tumefaciens* GV2260 (pBBRMCS1-5, pIG121-Hm); V1-G: *A. tumefaciens* GV2260 (pBBRacdS, pIG121-Hm); V3-G: *A. tumefaciens* GV2260 (pBBRgabT, pIG121-Hm); V4-G: *A. tumefaciens* GV2260 (pBBRacdSgabT, pIG121-Hm).
