# Supplementary figures and images for: Super-Agrobacterium ver. 4: Improving the Transformation Frequencies and Genetic Engineering Possibilities for Crop Plants
Source: Front Plant Sci. 2019 Oct 7;10:1204. doi: 10.3389/fpls.2019.01204 (PMC6791131; doi:10.3389/fpls.2019.01204)

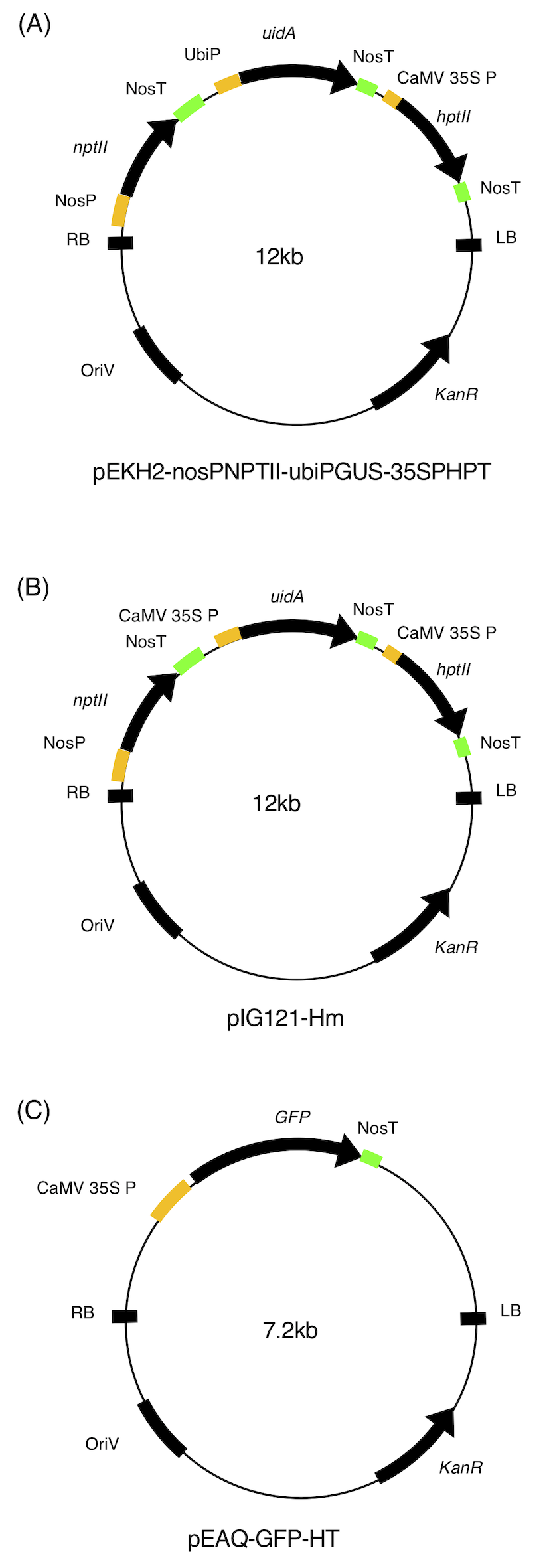

Supplement: Supplemental Figure 1 — Map of binary vectors. (A) Map of pEKH2-nosPNPTII-ubiPGUS-35SPHPT. NosP; Nopalin synthesis gene promoter, UbiP; ubiquitin gene promoter from rice, NosT; Nopalin synthesis gene terminator, nptII; neomycin phosphotransferase gene, uidA; beta-glucuronidase gene, hptII; hygromycin phosphotransferase gene, OriV; replication origin V (IncPα, plasmid RK2 from E. coli, GeneBank accession #J01780), KanR; Kanamycin resistance gene. RB; Right border sequence, LB; Left border sequence. (B) Map of pIG121-Hm. NosP; Nopalin synthesis gene promoter, CaMV 35S P; Cauliflower mosaic virus 35S promoter, NosT; Nopalin synthesis gene terminator, nptII; neomycin phosphotransferase gene, uidA; beta-glucuronidase gene, hptII; hygromycin phosphotransferase gene, OriV; replication origin V (IncPα, plasmid RK2 from E. coli, GeneBank accession #J01780), KanR; Kanamycin resistance gene, RB; Right border sequence, LB; Left border sequence. (C) Map of pEAQ-GFP-HT. CaMV 35S P; Cauliflower mosaic virus 35S promoter, NosT; Nopalin synthesis gene terminator, GFP; green fluorescence gene, RB; Right border sequence, LB; Left border sequence. [file Image_1.tiff]

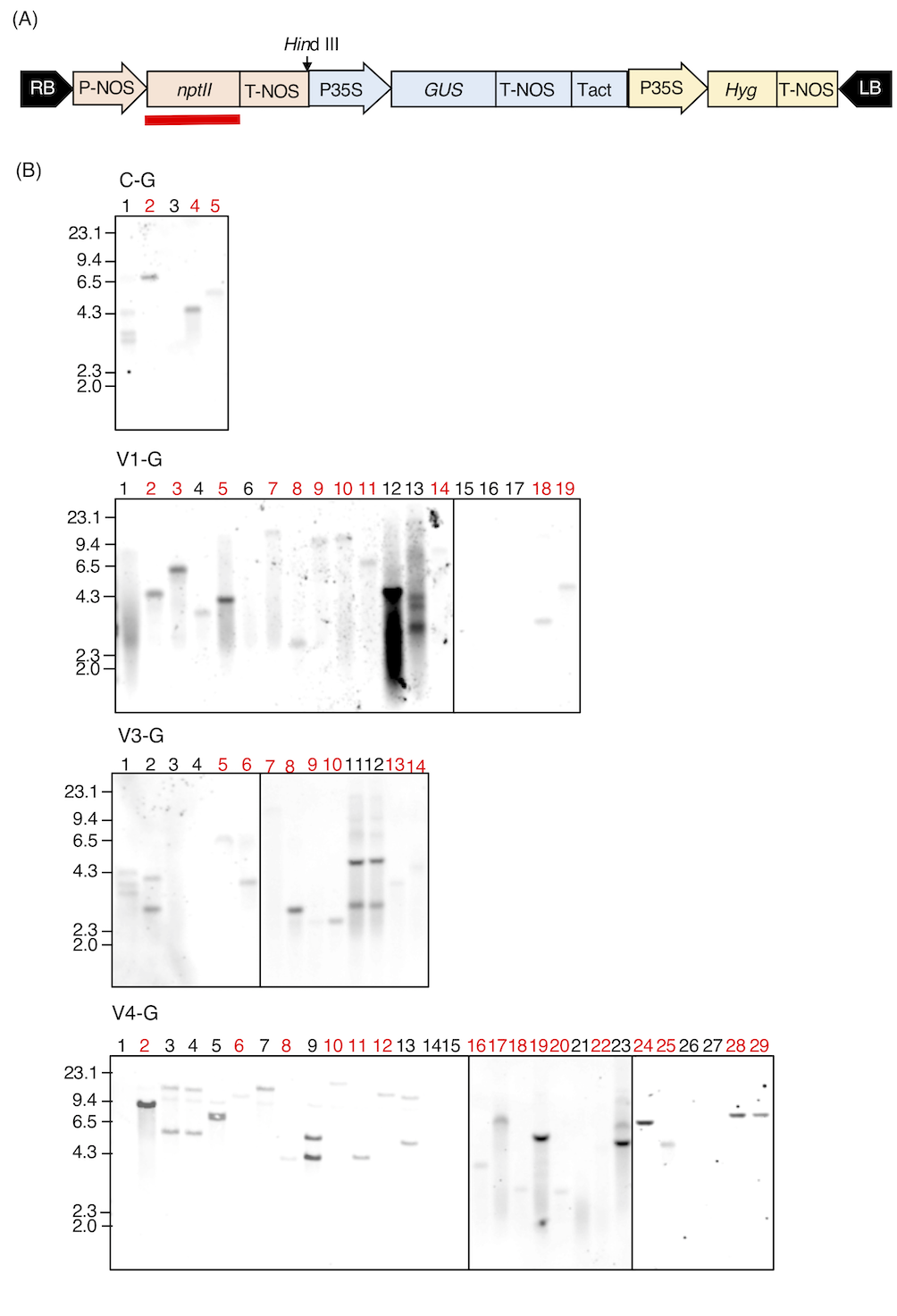

Supplement: Supplemental Figure 2 — Map of the pIG121-Hm vector and the southern blot analysis of the T0 generation. (A) Present the maps of the T-DNA region in the pIG121-Hm expression vectors used for the stable transformation. Red bars represent the position of probes used in the southern blot analysis. HindIII indicates the restriction enzyme sites that were used in the southern hybridization. NPTII probes were used in. (B) Southern blot analysis of the T0 generation. Red numbers indicate the transgenic lines with single copy. C-G: A. tumefaciens GV2260 (pBBRMCS1-5, pIG121-Hm); V1-G: A. tumefaciens GV2260 (pBBRacdS, pIG121-Hm); V3-G: A. tumefaciens GV2260 (pBBRgabT, pIG121-Hm); V4-G: A. tumefaciens GV2260 (pBBRacdSgabT, pIG121-Hm). [file Image_2.tiff]

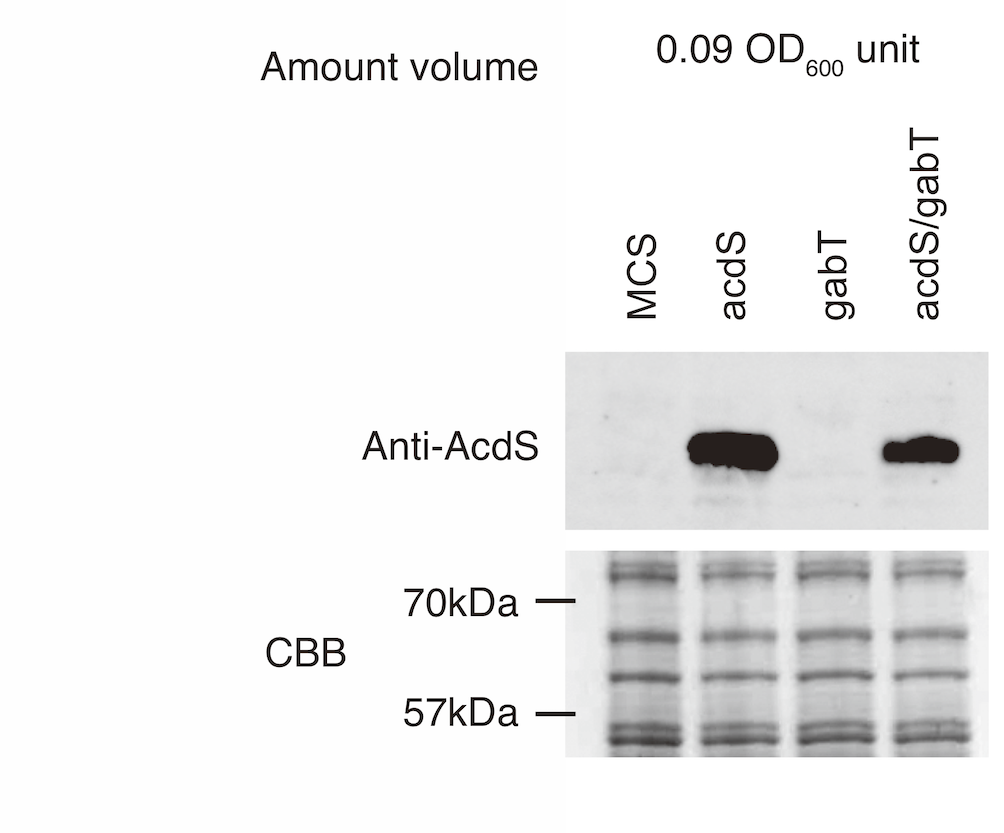

Supplement: Supplemental Figure 3 — Western blot analysis of ACC deaminase expression in A. tumefaciens strains using the cell lysate at the ‘Early stage’ (O.D.600 0.7). ACC deaminase was probed with an anti-ACC deaminase antibody. Coomassie Brilliant Blue staining (bottom panel) is shown as an internal control. C-G: A. tumefaciens GV2260 (pBBRMCS1-5, pIG121-Hm); V1-G: A. tumefaciens GV2260 (pBBRacdS, pIG121-Hm); V3-G: A. tumefaciens GV2260 (pBBRgabT, pIG121-Hm); V4-G: A. tumefaciens GV2260 (pBBRacdSgabT, pIG121-Hm). [file Image_3.tiff]
